# Cloud of Things in Crowd Engineering: A Tile-Map-Based Method for Intelligent Monitoring of Outdoor Crowd Density

**DOI:** 10.3390/s22093328

**Published:** 2022-04-26

**Authors:** Abdullah Alamri

**Affiliations:** College of Computer Science and Engineering, University of Jeddah, Jeddah 23890, Saudi Arabia; amalamri@uj.edu.sa

**Keywords:** outdoor localization, crowd safety, crowd monitoring, mobility, thermal cameras, cloud of things

## Abstract

Managing citizen and community safety is one of the most essential services that future cities will require. Crowd analysis and monitoring are also a high priority in the current COVID-19 pandemic scenario, especially because large-scale gatherings can significantly increase the risk of infection transmission. However, crowd tracking presents several complex technical challenges, including accurate people counting and privacy preservation. In this study, using a tile-map-based method, a new intelligent method is proposed which is integrated with the cloud of things and data analytics to provide intelligent monitoring of outdoor crowd density. The proposed system can detect and intelligently analyze the pattern of crowd activity to implement contingency plans, reducing accidents, ensuring public safety, and establishing a smart city. The experimental results demonstrate the feasibility of the proposed model in detecting crowd density status in real-time. It can effectively assist with crowd management tasks such as monitoring, guiding, and managing crowds to ensure safety. In addition, the proposed algorithm provides acceptable performance.

## 1. Introduction

The efficient management of citizen and community safety is one of the most important services required for future cities. In particular, the management of safety, specifically crowd management, is crucial during large-scale outdoor gatherings, such as those at beaches, public parks, gardens, or public open spaces, or at sport, entertainment, or religious events. In these spaces, it is important to monitor, guide, and manage large groups of people to ensure their safety.

Crowd management is a difficult undertaking that has an impact on how public spaces operate. In particular, analysis and monitoring are high priorities in the current COVID-19 pandemic scenario. Even more so, following an initial lock-down period during the pandemic, communities are still unsure how to resume normal life while the virus is still circulating in the community [1,2].

Large-scale gatherings can significantly increase the danger of transmission of infection. Hence, there is a vital need to develop a real-time crowd monitoring system that can detect and intelligently analyze patterns of crowd activity in order to implement effective contingency plans, reduce accidents, ensure public safety, and establish a smart city. With an intelligent crowd-monitoring system capable of monitoring a crowd’s movement, it will be easier to maintain social distancing safety in small and large public areas. Maintaining a minimum physical distance (also known as “social distance”) has been one of the most important means of disease control implemented by many nations worldwide. The main question here is how to follow social distancing policies and limit the size of gatherings in open public spaces. In this research, we propose a new method for the intelligent monitoring of outdoor crowd density using a cloud of things and analytics. This study focuses on crowd monitoring in static locations; that is, when people congregate at a certain spot or when a crowd moves from one place to another.

However, the tracking of crowd movement faces several complex technical challenges, particularly in terms of accurate head counting and the preservation of privacy. The design and application of sensors and procedures must be such that individual privacy is assured at the same time that accurate information is provided about the crowd density at the location.

The Internet of Things (IoT) has greatly improved numerous applications such as healthcare systems, indoor tracking, and mobility monitoring in urban environments by combining communication technologies and data analytics [3]. In addition, data from mobile devices has received a lot of interest as human mobility becomes more essential in many smart-city applications, such as the global positioning system (GPS), which has been the most extensively utilized technology for outdoor positioning. In this work, IoT is integrated with cloud and GPS-based mobility to improve crowd tracking and produce accurate crowd size estimations.

In summary, the contributions of the research described in this study can be listed as follows:

- A highly-structured intelligent method integrated with the cloud of things and data analytics for monitoring outdoor crowd density.
- A map tile mechanism for open outdoor spaces that divides the map area into multiple map tiles to adjust the social distancing in the outdoor area and control the density of moving objects in a given location.
- A crowd data collection and monitoring system for the tracking of crowd movement and estimation of crowd size. The proposed system combines GPS-based mobility and thermal cameras. This addresses the challenge of obtaining accurate crowd density estimates, as the shortcomings of one technology are compensated for by the features of the other.

The remainder of the study is organized as follows: Section 2 explains the background and current works relevant to this field of study. Section 3 explains the architecture of the proposed structure including crowd data collection and monitoring, outdoor map tile mechanism, and cloud IoT big data processing and analytics. Section 4 describes the experiments and presents the results of the evaluation of the proposed algorithms. Section 5 concludes the study with suggestions for future research directions.

## 2. Background and State of the Art

In recent years, a variety of crowd monitoring systems have been developed, and a variety of sensor types have been used to provide input to them. This section gives an overview of the most common sensor types used in crowd monitoring systems, as well as their shortcomings. Crowd mobility analytics is concerned with determining the distribution of people and their movements in certain locations. In general, computer vision-based approaches, RFID sensors, automatic counting systems, Bluetooth and WiFi sensors, and GPS sensors are some of the monitoring tools that offer real-time information about crowd movements.

Computer-based approaches make the classification based on features learned from photos or videos to discover people. Reference [4] proposed using an optimal neural network for improving the detection accuracy and speeds in order to estimate crowd density. References [5,6] proposed a mechanism to identify the object from images or video feed taken from different cameras. Reference [7] proposed a mechanism to detect dense crowds in images. Reference [8] proposed image processing approaches that can assist both data collection and online crowd monitoring, utilizing current closed-circuit television systems (CCTV). Reference [9] presented a tool that detects abnormal situations in crowd movement, using optical flow algorithms. Reference [10] presented a method for estimating crowd density based on variations in texture patterns in crowd photos. Several computer vision-based methods were presented in [11], although they may compromise personal privacy. Furthermore, this sort of crowd monitoring system requires a substantial quantity of support infrastructure (i.e., electricity and communication infrastructure), which might be difficult to establish.

RFID (Radio Frequency Identification) sensors, both active and passive, are frequently utilized at sporting, entertainment, and religious events [12,13]. Reference [14] developed an RFID system for tracking pilgrims by using the RFID tag placed inside a mobile phone and using the Internet to send location information to a server managed by the Hajj authority. Apart from RFID, crowd identification and human motion tracking can be done through WiFi and Bluetooth sensors, which capture communication signals transmitted over WiFi/Bluetooth. Reference [15] presented a method for estimating travel time from data gathered by Wi-Fi sensors. Reference [16] provided a system for crowd behavior analysis using non-invasive Wi-Fi probes. In Reference [17], Wi-Fi sniffers were used at an industrial show to capture WiFi probes from participants’ mobile devices, and mobility habits in each monitored zone were examined. Reference [18] presented a system that uses WiFi sensors to detect people flow based on a series of frequently visited sensing zones. WiFi sniffer technology has been used for crowd detection and monitoring in certain industrial products [19,20]. The shortcoming of using RFID is that the range of sensing stations is relatively small. In addition, Wi-Fi and Bluetooth sensors require strict digital security measures, which are inevitable to ensure public privacy and to identify and record the MAC address of Wi-Fi-enabled devices in their vicinity.

Other digital sensors, such as thermal cameras [21] and depth sensors [22,23], have recently been developed that can count pedestrians. The primary benefit of these modern counting systems is that they inherently respect pedestrian privacy. According to the literature, GPS trackers have been utilized for determining walking speeds, visitors’ routes, and activity zones [24]. Similarly, smartphone applications have been used to control crowds at the Hajj [25] and track traffic at music festivals [26].

In this study, the tile-map-based method for intelligent monitoring of outdoor crowd density offers several advantages. First, the outdoor area is divided into map tiles, according to the requirements of the place, to adjust the social distancing in the outdoor area and to control the density of moving objects in a given place. Second, it makes use of the GPS for crowd monitoring purposes. The growth of the mobile phone industry has resulted in powerful phones that are now equipped with a variety of sensors, including GPS localization. Privacy issues with GPS devices are typically minimal as users explicitly consent to their movements being tracked. Third, the key significant technological advance is the ability to combine both GPS-based mobility and IoT digital sensors, as seen in thermal cameras. This addresses the challenge of obtaining accurate crowd density estimates, as the shortcomings of one technology are compensated for by the features of the other. Finally, the proposed system is integrated with the cloud of things and data analytics to provide intelligent monitoring of outdoor crowd density using a tile-map-based method.

## 3. Cloud of Things in Crowd Engineering

**Figure 1:**
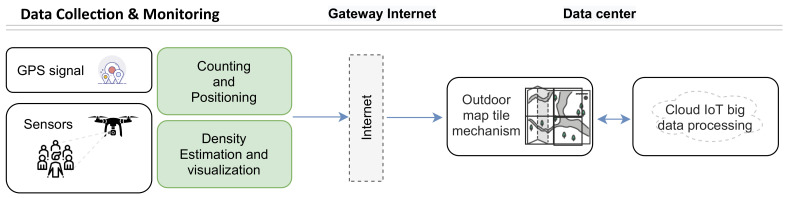
The task automation of intelligent monitoring of outdoor crowd density system.

The details of the proposed system for intelligent monitoring of outdoor crowd density are presented in this section. Task automation is enabled by the system illustrated in Figure 1 and by procedures based on data collected by the distributed sensors and GPS-based mobility. The construction of the proposed approach involves the following: crowd data collection and monitoring, outdoor map tile mechanism, and cloud IoT big data processing and analytics. Technically, the system will gather crowd position data, using GPS status and sensor assistance methods, and then build up a repository in a cloud server through the Internet service provider. Using our outdoor map tile technique, the server partitions the outdoor map into map tiles. In the final phase, data analytics and the cloud of things are utilized to determine crowd density. Each step is described in detail in the following sections.

### 3.1. Crowd Data Collection and Monitoring

For crowd movement tracking, and accurate crowd size counts, various techniques are used for data generation and processing.

**Figure 2:**
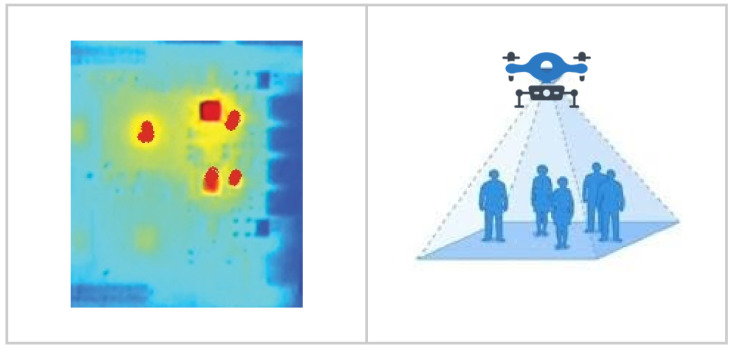
Example of thermal imaging system based on a temperature sensor array.

- GPS: In open spaces, the GPS data module is one of the best means of estimating outdoor crowd sizes and observing pedestrian movements in a certain location. Smartphones have become one of humanity’s most ubiquitous technologies, as millions of people now carry them and, therefore, they are equipped with assisted GPS in their hands or pockets. A GPS utilizes smartphone networks in conjunction with a GPS antenna to improve the speed of identifying or fixing positions. However, in some cases, a GPS cannot operate in a location where there is no network coverage or if the user does not collect his/her own location information.
- Infrared Thermal Imaging System: The number of people in a location can be determined by thermal image processing. It detects the density of the crowd in a particular location via infrared radiation detection, which is triggered by body heat. Figure 2 shows an example of a thermal imaging system based on a temperature sensor array to detect heat sources. Because thermal radiation penetrates smoke, aerosols, dust, and mists more efficiently than it does visible radiation, it is superior to visible imaging methods for detecting crowds in a wide variety of normally troublesome atmospheric conditions. Table 1 summarizes the pros and cons of employing RGB and thermal cameras to detect and track people.

**Figure 3:**
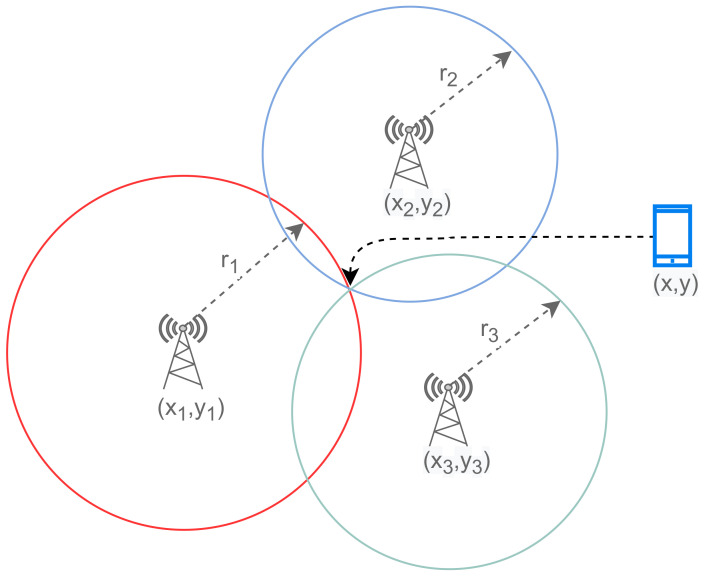
Trilateration used by GPS receivers.

To determine the crowd density in a location, the data collected by GPS devices in the area are utilized. GPS positioning is based on trilateration, which is a technique for estimating position by measuring distances between points with known coordinates [27,28,29,30]. Trilateration calculations use the coordinates (longitude, latitude) of neighboring cell towers, as well as the estimated distance between the device and the cell towers, to determine the coordinates of a mobile device. Figure 3 shows the trilateration process used by GPS receivers.

**Definition** **1.** *Data detection is represented as tuple $T_{i}$ = ($D_{id}$, $t_{i}$, $l_{i}$) where:*

- *$D_{id}$ is the device ID whose location will be tracked.*
- *$t_{i}$ is the timestamp showing when the user device entered or exited the zone.*
- *$l_{i}$ is the latitude and longitude used to determine the user’s precise position within the zone (spatio-temporal data of the user).*

Anomalies/outliers in GPS data, which affect positioning precision, are an example of sensor defects. The GPS signals may be affected by multipath error or obstructed in urban areas in some circumstances. To address this issue, thermal image sensors can also be used to provide detection of objects and count and to alleviate difficulties caused by a lack of GPS signals. Compared with conventional image cameras, the superior capabilities and accuracy of thermal imaging have led to the use of thermal cameras in people-counting and tracking applications, particularly because thermal imaging uses a non-intrusive passive sensor that also preserves privacy.

The proposed system consists of GPS-based mobility and thermal cameras that work together to compensate for each other’s main shortcomings. GPS-based mobility provides a low-cost and preserving solution for crowd tracking and monitoring. Based on captured GPS data, the system can count mobile devices in target tiles. Thermal cameras can provide higher accuracy and optimized counts based on precise near-ground truth. The key idea of the proposed cross-crowd detection approach is to form a database that stores the correlation between the number of people and density captured by GPS-based mobility and the number of people counted by the thermal camera with anomaly detection in GPS data. Both GPS-based detection and thermal camera counts are reported to the cloud, where data analytics modules reside. The analytical results are applied based on the correlation between the two data modalities.

### 3.2. Outdoor Map Tile Mechanism

**Figure 4:**
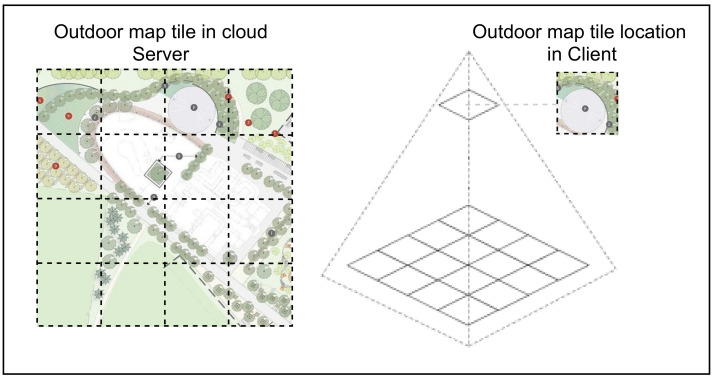
Outdoor map tile mechanism.

In this work, in order to determine the maximum number of people gathering at the same time at a specific location in an outdoor space, we present a map tile mechanism for open outdoor space as represented in Figure 4. The basic idea is to adapt social distancing in the outdoor portion in order to control the density of moving objects in a certain location.

In the map tile mechanism, outdoor space is split into map tiles depending on the requirements of the location (Definition 2). Each tile contains X and Y according to the location (longitude and latitude) of uploaded data in the server. A client-side local database stores the map tile *m*${}_{ti}$ as well. Based on GPS-based mobility, the purpose of utilizing the user device is to verify that the user is roughly situated in the map tile *m*${}_{ti}$ in the local database.

In addition, data captured by the thermal image is decomposed into *∂* blocks. Each block is then analyzed to calculate the density of moving objects in a certain block. The data collected from both GPS-based mobility and sensor assistance methods results in a build up of a repository in a cloud used to determine crowd density status.

**Definition** **2.** *The outdoor map area O${}_{m}$ is divided into multiple map tiles (O${}_{m}$ = m${}_{t1}$, m${}_{t2}$, …, m${}_{tn}$; i = 1, …, n) where m${}_{ti}$ indicates a specific map tile. Let S be the square area of an outdoor tile; then, the length of each tile is set to m${}_{ti}$$\overset{}{\to }$ S.*

**Definition** **3.** *Assume that thermal image I is broken down into ∂ blocks. Consider the ∂${}_{i}$ bloc and crowd density d as the following: ∂${}_{i}$→ I${}_{d}^{\partial }$, where I${}_{d}^{\partial }$ represents the crowd density values of the target block.*

**Figure 5:**
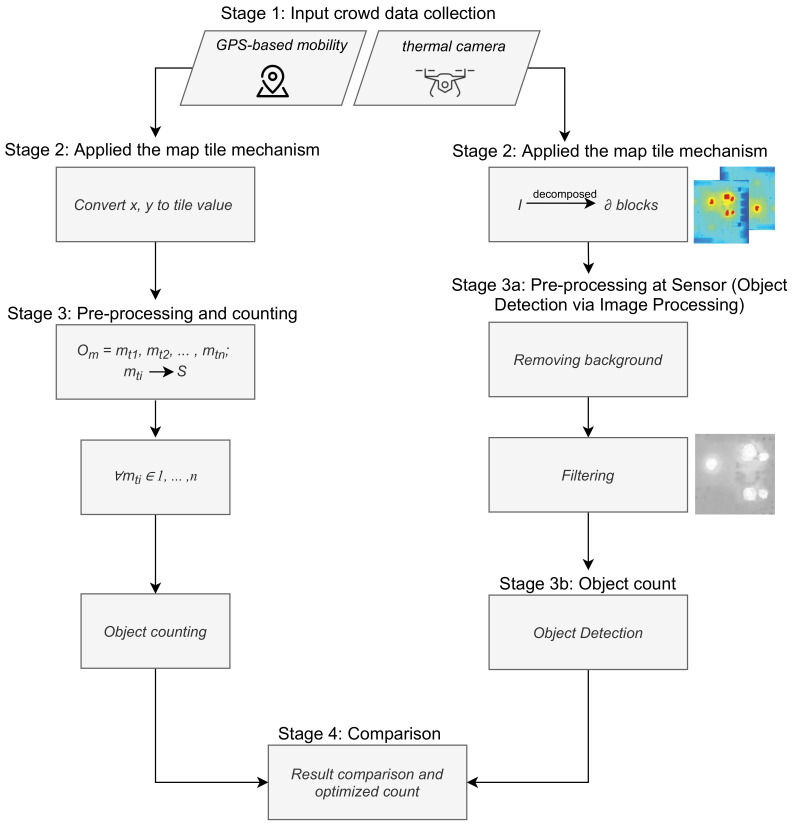
The schematic diagram of the proposed crowd mobility tracking and crowd size counts.

The schematic diagram of the proposed crowd mobility tracking and crowd size counts is illustrated in Figure 5. It shows the stages of the crowd-density assessment of a location using GPS status and the proposed thermal distribution of the crowd monitoring system. As shown in Figure 5, the GPS-based mobility counting stages convert X and Y according to the location (longitude and latitude) into tile values. In each map tile, the number of people and the density are extracted based on GPS status. The thermal distribution of the crowd monitoring system (Figure 5) shows the stages involved in the thermal counting of people. The thermal image is first upsampled, which increases the size and quality. After that, the foreground image is subtracted from the background image. The equalized image is converted to binary. The blob detection method is applied (Definition 4) to the foreground image to separate the merged blobs. It can help to distinguish people passing close to each other [31]. Finally, we extract the detection results, and the number of people and density are displayed.

**Definition** **4.** *Laplacian of Gaussian (LOG) is applied in the $\partial_{i}$ block to detect blobs and identify the possible crowd density values of the target block.*

### 3.3. Cloud IoT Big Data Processing and Analytics

Our cloud-based system for the intelligent monitoring of crowds has a number of advantages. To create an effective infrastructure, more than mere sensors and an Internet connection are required. Indeed, a system capable of collecting, storing, analyzing, processing, and managing the huge amount of intelligent data created is required to support this infrastructure (the big data challenge). Accordingly, the cloud-based system can handle massive data storage and intensive data processing and analysis tasks. It can also provide users with real-time information on the crowd density in a specific location, and it enables them to make well-informed and timely decisions.

The data from the bottom layer is modeled using the outdoor map tile mechanism to build up a repository in a cloud. The data in the cloud infrastructure is evaluated to determine the crowd density in a location. Based on the two data collections (GPS or thermal image) that were decomposed into tiles, the calibration of the data analytical results is applied.

**Figure 6:**
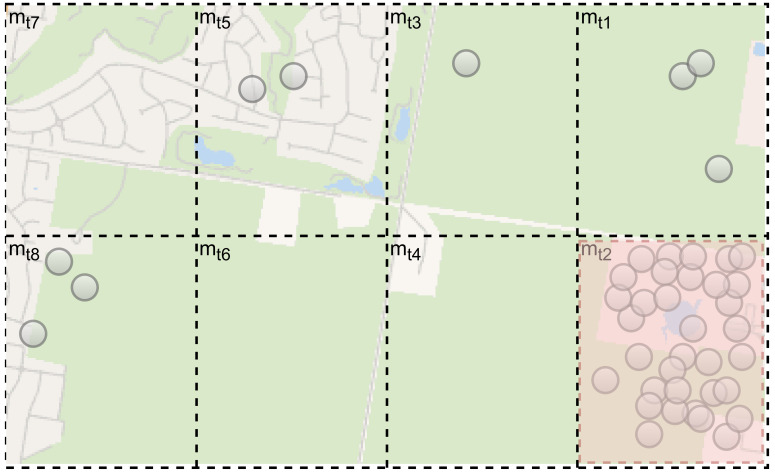
An example: *m*${}_{ti}$ = 36 square meters long, *D*${}_{max}$≤ 18.

The outdoor square size rule is adopted in this framework as it has been applied in different countries to control crowd density in outdoor spaces. In general, the maximum number of people allowed in an indoor space is one per 4 square meters, and for outdoor areas it is two per 2 square meters [32,33]. For example, if an outdoor space tile is 8 square meters long, and it has a density quotient of 2, then no more than four moving objects (people) should be in the outdoor space at the same time. Figure 6 shows an example of the square meter rule applied to an outdoor space area.

**Definition** **5.** *Let us consider S outdoor square size; the maximum density of moving objects u at t${}_{i}$ time can be calculated as:*

$$D_{max}=S/2.$$

**Figure 7:**
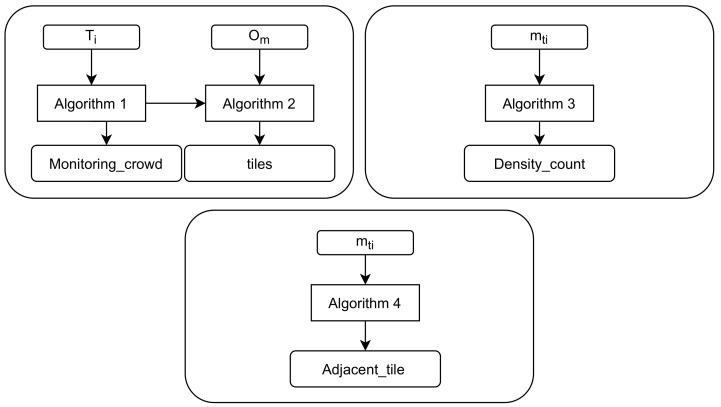
The proposed model is composed of multiple algorithms: monitoring crowd, estimating density count, and evaluating adjacent tile.

One of the properties of map tiles is that they can be represented differently at different scales. Because tile size varies, the calculation of crowd density usually depends on the tile size. In this study, a tiling map will divide the map into several tiles of a fixed size. Assume that the moving objects in a certain tile *m*${}_{ti}$ have exceeded the allowed density based on Definition 5. The tile *m*${}_{ti}$ will be “restricted” and no new objects should be allowed in until the tile has been “released”. The users’ devices located in the map tile *m*${}_{ti}$ will receive an alarm message that the *m*${}_{ti}$ has the maximum capacity of moving objects and is restricted. In addition, it identifies the moving objects that have spent a long time in that tile and advises them to exit or to move to a tile that has no restrictions. Furthermore, users within a certain radius around restricted tiles receive notifications of the crowd density status of each map tile. Figure 7 shows the model proposed in this study, which consists of multiple algorithms, each with a distinct objective. The intelligent monitoring of outdoor crowd density can be summarized as the pseudo-code shown in Algorithm 1. The outdoor map tile is shown in Algorithm 2. The density rule is shown in Algorithm 3, and the evaluation of the moving objects with time to exit or move to another tile is shown in Algorithm 4.
**Algorithm 1:** Live outdoor monitoring density algorithm.
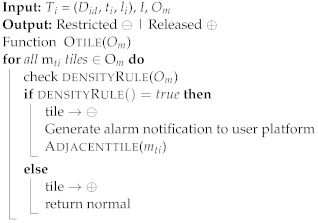

**Algorithm 2:** Outdoor map tile algorithm.
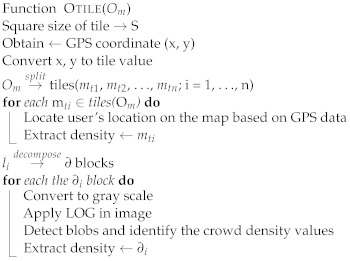

**Algorithm 3:** Density algorithm.
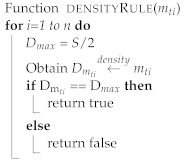

**Algorithm 4:** Adjacent tile algorithm.
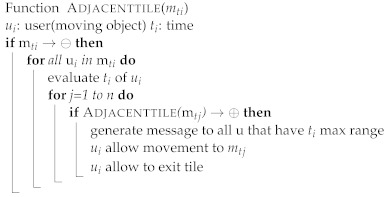


## 4. Performance Analysis and Simulation

In this section, the experimental results obtained after evaluating the proposed intelligent monitoring model are presented. The technique was implemented in Python, and the data were stored using the MongoDB distributed database. MongoDB is also connected with Apache Spark which has greater real-time analytics and machine-learning capabilities. The implementation of a mobile application is outside the scope of this study.

**Figure 8:**
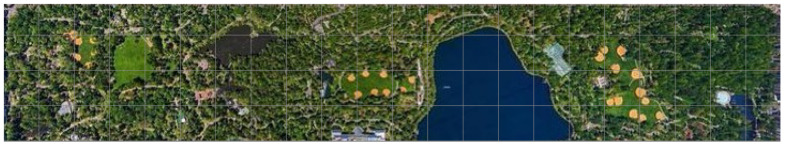
Example of outdoor environment.

In this experiment, we used an actual outdoor environment: Central Park in New York City (Figure 8). We generated the GPS data of moving objects synthetically based on realistic scenarios at random locations within the park. Furthermore, before beginning the experiment, the outdoor map area *O*${}_{m}$ was divided into multiple map tiles (*O*${}_{m}$ = *m*${}_{t1}$, *m*${}_{t2}$, …, *m*${}_{tn}$; *i* = 1, …, *n*) and we set *S*, the square area of an outdoor tile, to *m*${}_{ti}$$\overset{}{\to }$ 500 m${}^{2}$.

**Figure 9:**
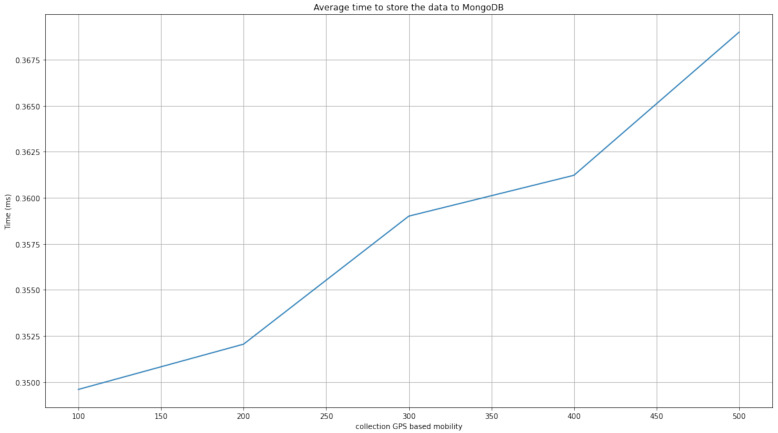
The average time to store the data to MongoDB.

**Figure 10:**
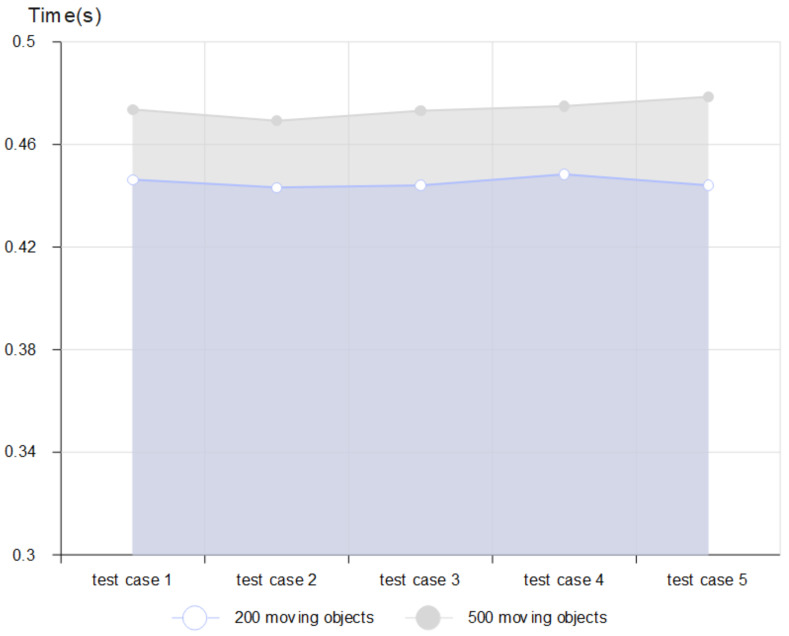
Random test cases (200 and 500 moving objects).

After the first phase in which the database was constructed, the GPS data of moving objects in our database were allocated to the corresponding map tile. Figure 9 shows the average time required to store the data to MongoDB from the moment that GPS-based mobility data were collected. We also evaluated the proposed method for the effective measurement of construction performance. We increased the number of moving objects and conducted random test cases of sufficient quantity to evaluate performance. Figure 10 show that the tile-map-based method for the intelligent monitoring of outdoor crowd density performs well. For example, the proposed model demonstrates a consistently stable and low response time in a test scenario which involves 500 moving objects.

**Figure 11:**
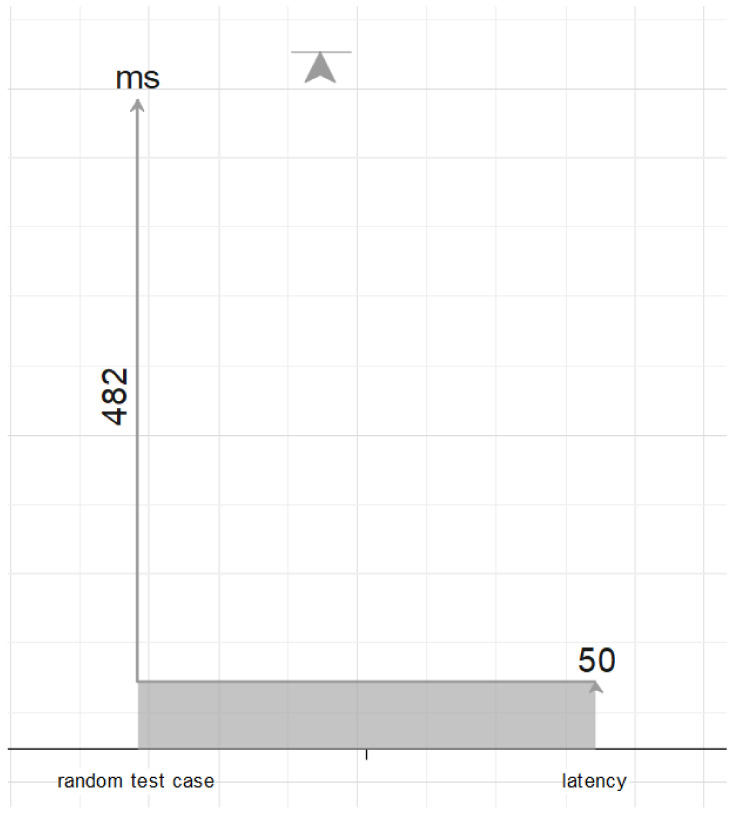
The construction costs of the proposed method, taking into account the latency of the network.

**Figure 12:**
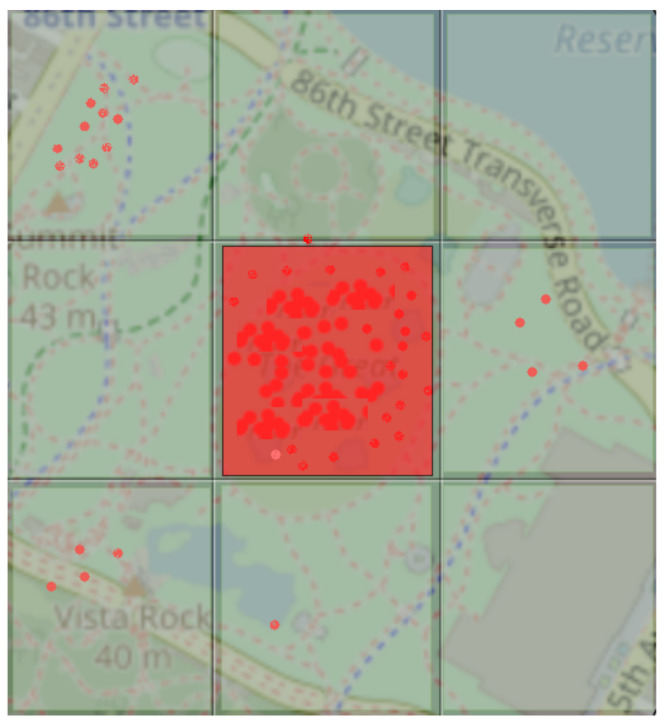
A simulation example at time t1 of the proposed model.

We also measured the construction costs of the proposed method, taking into account the latency of the network. In fact, with a density of 500 objects and a conventional 4G latency of 50 ms for delivering data and warnings, our system can send an alert notification in 532 ms, as illustrated in Figure 11. In addition, we provide some examples of the simulation of the proposed model. Figure 12 depicts a simulation of the proposed model for 200 objects at time point t1.

The evaluation results show the feasibility of the proposed model in detecting crowd density status in real-time, indicating that it can effectively assist with crowd management tasks such as monitoring, guiding, and managing crowds in order to ensure safety. Furthermore, it is obvious that when the density of moving objects in different circumstances increases, the proposed algorithm can still perform well in terms of construction costs.

While the results presented in this study provide acceptable performance, we recognize that the experience needs to be enhanced, which will serve as a foundation for future studies. A comparison with similar approaches will be presented. In addition, through real-world deployment, we aim to evaluate its effectiveness and performance. In addition, we aim to investigate the accuracy of our methodology by comparing our crowd density estimates to ground truth information.

## 5. Conclusions

In this study, using a tile-map-based method, we proposed a highly-structured intelligent system integrated with the cloud of things and data analytics for monitoring outdoor crowd density. From a functional perspective, the construction of the proposed approach structure involves the following: crowd data collection and monitoring, outdoor map tile mechanism, and cloud IoT big data processing and analytics. An intelligent crowd-monitoring system that is capable of monitoring the crowd movement makes it easier to maintain social distancing safety in small and large public areas. The results reveal that the proposed model can identify crowd density status in real-time, implying that it can effectively assist crowd management tasks such as monitoring, guiding, and managing crowds to ensure safety.

The experiment described in this study will be improved in the future, and findings for larger data sets will be obtained. Future work on actual experiments will be used to develop this framework for practical use.

## Figures and Tables

**Table 1:** The benefits and drawbacks of utilizing RGB and thermal cameras to detect and track people.

	Benefits	Drawbacks
RGB	Not expensive Re-identification possible	Privacy problems Shadows light sensitivity
thermal cameras	No privacy issues Unaffected by light Easier segmentation	More expensive Reflections

## Data Availability

The experimental data are contained within the article.
